# Modulation of Reactive Oxygen Species in Health and Disease

**DOI:** 10.3390/antiox8110513

**Published:** 2019-10-26

**Authors:** Solomon Habtemariam

**Affiliations:** Pharmacognosy Research Laboratories & Herbal Analysis Services UK, University of Greenwich, Chatham-Maritime, Kent ME4 4TB, UK; s.habtemariamn@herbalanalysis.co.uk; Tel.: +44-208-331-8302/8424

In diverse living organisms, signaling within the cell, chemical communication between cells or simply the fate of cells to survive or die is largely dependent on the intricate balance of control mechanisms related to reactive oxygen species (ROS). Low or basal levels of ROS are generally considered indispensable for the normal physiological function, while excessive levels, either due to increased ROS production or weakened antioxidant defenses, are implicated in a number of diseases ranging from infection and inflammation to chronic cardiovascular, neurodegenerative, and metabolic disorders. Understanding the state of ROS-based pathology (or oxidative stress, OS) and intervention mechanisms are thus crucial in the potential management of a plethora of human diseases. Against this background, the special issue “Modulation of Reactive Oxygen Species in Health and Disease” was designed to shade some novel insights into the role of ROS as disease modulators, pathological markers and their targeting by potential therapeutic agents (Figure 1).

One of by far the most studied and established mode of action for the deleterious action of ROS in biological systems is direct damage to cellular macromolecules such as proteins, lipids and DNA. The resulting cell damage or death induced by either necrosis or apoptotic mechanisms could lead to functional losses and pathologies. Rugemalira et al. [1] studied the role of such pathological alterations in the cerebrospinal fluid of paediatric patients due to bacterial meningitis. As a marker of OS changes, the protein and DNA oxidation assessed where ortho-tyrosine, 3-chlorotyrosine, 3-nitrotyrosine and 8-oxo-2′-deoxyguanosine. The association of OS with childhood bacterial meningitis was evident given the oxidised levels of these biomarkers were by order of magnitude elevated in these patients. Moreover, the intertwining pathway of inflammation and oxidative stress was also evident in pneumococcal meningitis as the myloperoxidase activation marker, 3-chlorotyrosine, was elevated [1]. A prospective case-control study in children with pneumococcal meningitis also revealed an increased level of expression for genes regulating immune activation, OS, leucocyte adhesion and/or migration [2]. These findings are also in line with the numerous studies from our laboratories and elsewhere that have shown the interlinking pathway of OS and inflammation in the pathogenesis of many chronic diseases.

A review article by Miyata et al. [3] outlined the role of OS in urinary dysfunction following bladder outlet obstruction. They have outlined that the pattern of ischemia and repeated ischemia/reperfusion leading to bladder damage has a significant OS component that could be targeted by antioxidant mechanisms as with other ROS-dependent pathologies. On this basis, the protective effects of ROS scavengers (e.g., epigallocatechin gallate) in OS associated with bladder outlet obstruction in experimental animals have also been demonstrated [4,5]. As with many other OS-associated diseases, the link between OS and inflammation in bladder outlet obstruction has further been established [6]. Another important development in the role of OS in human diseases came from studies on human patients with uterine polyp, myoma, hyperplasia, and adenocarcinoma [7]. On the basis of the levels of lipid peroxidation and altered uterine antioxidant enzymes (e.g., copper–zinc superoxide dismutase, catalase, glutathione peroxidase, glutathione reductase, and nuclear factor erythroid-2-related factor 2 transcription factor (Nrf2)), the role of diverse redox mechanisms in etiopathogenesis of different gynecological diseases were established.

The association between the activity of the gene encoding the matrix thiol oxidoreductase, glutaredoxin-2 (GRX2), and mitochondrial bioenergetics was studied by Mallay et al. [8]. They have shown that the deletion of this gene could enhance the proton leak-dependent and phosphorylating respiration in mitochondria isolated from the liver of female, but not male mice. On the other hand, deletion of this gene could augment proton leaks and ADP-stimulated respiration in muscles in male but not female mice. Hence, the production of ROS production such as H_2_O_2_ in various tissues could be dependent on sex suggesting possible gender-based variations in the pathology of OS-dependent disease. Considering the wider role of GRX2 in human cardiac pathologies [9], brain injury [10], eye diseases [11], etc., its gender-dependent expression in the various tissues is worth further investigation.

One of the most ubiquitous antioxidant systems in the body is based on the regulation of the level of reduced (GSH) and oxidised (GSSG) forms of glutathione. As a main non-enzymatic antioxidant, GSH directly scavenge ROS. The study by Kwon et al. [12] further showed the role of GSH in macrophage polarization-mediated immune response. Beyond an antioxidant, GSH could boost the immune response by increasing the levels of pro-inflammatory cytokines (interleukins and tumour necrosis factor-α), neutrophil-attracting chemokines, nitric oxide and prostaglandin E_2_. This inevitably activated the nuclear factor kappa B or macrophages, while ROS generation induced by bacterial lipopolysaccharide was suppressed. In this connection, the antioxidant activity of many natural products have been shown not only through direct scavenging effect on ROS but also via induction of antioxidant defences primarily through the Nrf2/ heme oxygenease-1 (HO-1) signaling pathway. Hence, non-phenolic compounds that are not known for direct ROS scavenging such as monoterpenes, triterpens including ursolic acid and ginsenosides have all shown to ameliorate OS in experimental models through induction of antioxidant defences (e.g., [13,14,15]). It is interesting to note that GSH as with many antioxidant natural products was also shown to ameliorate ROS via the Nrf2-dependent HO-1 which is also implicated in immunostimulation as evidenced from the regulation of M1-like macrophage polarization [12]. 

Melatonin being a natural antioxidant that received a lot of attention in recent years, Espino et al. [16] studied its level in follicular fluid and potential role in the regulation of human fertility. Their most significant finding was the association between low level of melatonin in women with unexplained infertility and that melatonin supplementation could ameliorate intrafollicular oxidative balance and oocyte quality. This effect was further shown to lead to a small increase in the rate of pregnancies [16]. In addition to the role of melatonin in delaying ovarian aging, ovarian turn-over and follicular development [17,18], it has been shown to increase human spermatozoal activity by ameliorating mitochondrial dysfunction [19] and under various other OS conditions [20,21,22].

Undoubtedly, most of the antioxidant compounds researched for potential therapeutic intervention come from natural sources, primarily plants. Polyphenolic compounds such as flavonoids as ROS scavengers and their plethora of pharmacological effects in OS-associated disease have been extensively investigated. One of these flavonoids is rutin which is the 3-*O*-rutinoside of quercetin. As a natural source of this bioactive molecule, the common buckwheat green (*Fagopyrum esculentum* Moench (Polygonaceae)) has been extensively studied though other sources including *M. stenopetala* as potential sources have been advocated. A case study on rutin production and level of the potential phototoxic, fagopyrin, composition of *F. esculentum* grown in the UK was researched in our laboratory [23]. Indeed, buckwheat as a high rutin yielding plant could serve as a commercial source of rutin or as a functional food for OS associated diseases provided that an efficient methodology is employed for removing fagopyrins. Hence, the various OS-dependent effects demonstrated for rutin including as therapeutic potential for chronic diseases such Alzheimer’s disease and diabetes [24,25] should also be assessed for *F. esculentum* leaves.

In conclusion, ROS and OS continued to be the most popular research topic in human diseases. Considering their role in disease mediation, as pathological markers and therapeutic targets, the search for novel intervention mechanism is likely to resolve some of the unmet challenges in chronic human diseases. 

## Figures and Tables

**Figure 1:**
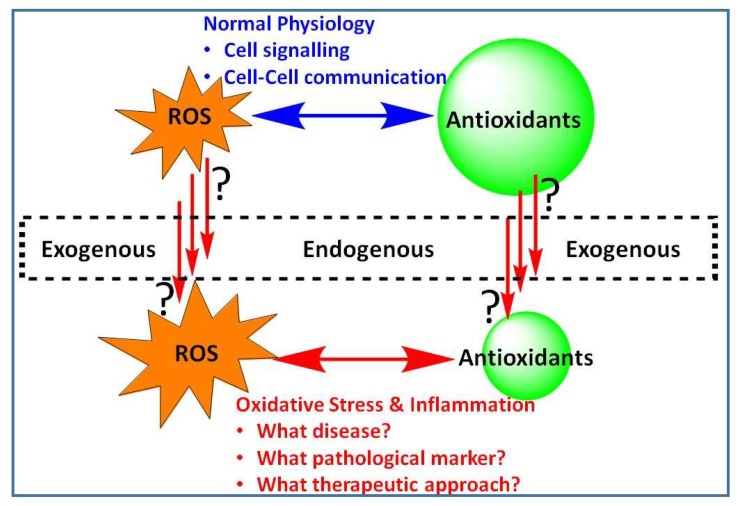
Reactive oxygen species (ROS) in health and disease. Generated under normal physiological conditions, ROS are vital to the normal functioning of cellular systems. Excessive level of ROS either from endogenous or exogenous sources; or that through increased level of their production or diminished antioxidant defences result in the development of oxidative stress (OS). This special issue focus on pathologies associated with OS and the interlinking inflammatory pathway with particular emphasis on disease markers and therapeutic intervention approaches.
